# Exploring the Typicality, Sensory Space, and Chemical Composition of Swedish Solaris Wines

**DOI:** 10.3390/foods9081107

**Published:** 2020-08-12

**Authors:** Gonzalo Garrido-Bañuelos, Jordi Ballester, Astrid Buica, Mihaela Mihnea

**Affiliations:** 1 Agriculture and Food, Product Design—RISE—Research Institutes of Sweden, 41276 Göteborg, Sweden; 2 Centre des Sciences du Goût et de l’Alimentation, AgroSup Dijon, CNRS, INRA, Univ. Bourgogne Franche-Comté, F-21000 Dijon, France; jordi.ballester@u-bourgogne.fr; 3 South African Grape and Wine Research Institute, Department of Viticulture and Oenology, Stellenbosch University, Stellenbosch 7600, South Africa; abuica@sun.ac.za; 4 Material and exterior design, Perception—RISE—Research Institutes of Sweden, 41276 Göteborg, Sweden

**Keywords:** Solaris, Sweden, hybrid grapes, typicality, sensory evaluation, chemical composition

## Abstract

The Swedish wine industry has exponentially grown in the last decade. However, Swedish wines remain largely unknown internationally. In this study, the typicality and sensory space of a set of twelve wines, including five Swedish Solaris wines, was evaluated blind by Swedish wine experts. The aim of the work was to evaluate whether the Swedish wine experts have a common concept of what a typical Solaris wines should smell and taste like or not and, also, to bring out more information about the sensory space and chemical composition of Solaris wines. The results showed a lack of agreement among the wine experts regarding the typicality of Solaris wines. This, together with the results from the sensory evaluation, could suggest the possibility of different wine styles for Solaris wines. From a chemical perspective, the global volatile profile showed a larger variability between individual wines than between Solaris and non-Solaris. However, 4MMP, ethyl propionate, ethyl 2-Methyl propanoate, and diethyl succinate were significantly higher in Solaris wines. Concerning non-volatile compounds, the results showed a significant discrimination between Solaris and non-Solaris wines, the former being characterized by higher ethanol %, Mg, succinic acid, tartaric acid, and sucrose levels.

## 1. Introduction

The international wine market is continuously evolving. In the past 50 years, wine production has increased, and the import and export markets have significantly expanded, partly influenced by the evolution of international trends in wine consumption [1]. The economic development of certain countries has definitely been a key factor in the revolution of the international wine industry [2] and global warming has been pointed out as a contributing factor [3]. On the one hand, climate change is affecting the typicality of wines from traditional wine regions [4] and, therefore, the regional viticulture strategies. In 2019, the Syndicat Viticole des Appellations d’Origine Controlée (AOC) Bordeaux and Bordeaux Supérieur allowed the plantation of new cultivars such as Marselan and Tinta Barocca for red grapes, and Albariño for white [5]. Moreover, the continuous rise in global temperatures is leading to the appearance of new wine-producing regions/countries, challenging the traditional classification of the “old world” (i.e., Spain, France, or Italy among other European countries) or the “new world” (i.e., South Africa, United States, Australia, or Argentina), as new wine-producing countries are emerging in areas such as the northern parts of Europe. Sweden is one of these countries. According to the latest report from Föreningen Svenskt Vin [6], Sweden had an approximate area under vine of 100 hectares at the end of 2017. First vineyard plantations started in 1999 and 20 years later, the Swedish wine industry is represented by more than 200 vineyards, out of which almost 30% have been planted in the past 5 years. The current projection estimates a continuous growing in the hectares surface, employees, and number vineyards to become a commercial business.

Climatic conditions are certainly more suitable for viticulture now than 20 years ago in Sweden; however, the cold temperatures and winter frost remain some of the biggest challenges. This is the main reason why the most cultivated and successful grapes are hybrid cultivars highly resistant to frost, such as the white variety Solaris and Rondo as a red grape cultivar [7]. Originating in Germany, Solaris (an interspecific hybrid result of the cross between Merzling (Seyve-Villard × Riesling × Pinot Gris) with Gm6493 (Zarya Seva × Muscat Ottonel)) [8], is the most planted grape cultivar in Sweden and Denmark. Besides its resistance to frost, Solaris is also highly tolerant to downy mildew [9,10], to oidium (powdery mildew), and to some extent also to Botrytis [11,12].

Despite its obvious agronomic interest, little information is available describing the aromatic potential using different winemaking techniques [13,14,15] or studies describing the Solaris sensory space [16,17,18]. For example, in Denmark, a study found Solaris wines with two different aroma profiles [18]; the first group of wines was described with floral and fruity flavors (“peach/apricot”, “Muscat”, “melon”, “banana” and “strawberry”), whereas the second group was associated with less pleasant flavors, such as “chemical”, “wood” and “rooibos/smoke” [18]. Another recent study aimed to assess the sensory similarity between three Solaris wines and three of the main international varietals, Riesling, Chardonnay, and Sauvignon Blanc, by means of Polarized Sensory Positioning [11]. The study concluded that Solaris wines were closer to Chardonnay and Sauvignon Blanc than to Riesling. A subsequent Pivot© Profile showed that Solaris wines were globally less viscous, sweeter, more vegetal, and more floral than Chardonnay, and less aromatic and fruity and more acidic and vegetal than Sauvignon Blanc. Finally, a study evaluating the sensory profile of Swedish Solaris described the wines with citrus and floral aromas [16].

The small number of studies on Solaris wines leads to inconclusive information regarding the existence of one or several styles of Solaris wines. This potential diversity of styles leads to the main question of the present work: Is there an existing concept of Solaris typicality? Do the Swedish wine experts have a shared representation of what a typical Solaris should smell and taste like? Therefore, it is necessary to investigate first if Solaris wines exist as a varietal wine sensory category and, if so, which are its most salient and typical sensory features.

Different approaches have been used to characterize wine categories from a sensory point of view. Some of those approaches were based on quantified sensory description and aimed to pinpoint specific co-occurrence of sensory features for a given wine category and not the neighboring categories [19]. From a cognitive point of view, typicality is the degree to which an item is a good example of its category [20]. This approach has been previously used to explore wine-related sensory categories such as variety [21] or origin [22,23]. Typicality of the wines is often associated to tradition as it is usually linked to a specific “terroir” or wine of origin, but it can also be linked to other parameters, such as the use of specific winemaking practices [24]. Additionally, the increase in temperatures due to climate change is defying the typicality of certain wine regions [4], leading to an adaptation of the viticulture and winemaking practices to preserve the typical character of the wines.

Several studies used alternative names to typicality such as exemplarity [25], representativity [26], or typicity [27]. The reliability of typicality measurements depends on the expertise or familiarity of the tasters with regard to the category at hand as well as on the between-judges agreement [21,22].

Often, typicality assessments are combined to sensory descriptive and physico-chemical analyses [22,23]. Different studies have investigated the chemical compounds, volatile and non-volatile, responsible for typical sensorial attributes of a number of relevant wine categories [19,28,29,30]. However, these compounds are not exclusive to one single grape variety, but their transformation during winemaking and the subsequent interactions with other compounds will contribute to the wine identity [31,32]. In this respect, very little information is available concerning Solaris varietal aromas and the non-volatile composition related to color, phenolic composition, acidity, or alcohol content [33,34,35]. Concerning primary-derived aromas, terpenes were previously reported in Danish Solaris [13] and a recent study has shown the presence of polyfunctional thiols in Belgian Solaris wines [36].

The present work aims to elucidate whether the Swedish wine experts have or not a preconceived idea on what a typical Solaris wine should smell and taste like. Together with Swedish Solaris wines, international white wines from different grape cultivars and origins were also investigated. In addition, the chemical characterization of the volatile and non-volatile composition of the wines aims to identify possible markers associated with Solaris wines.

## 2. Materials and Methods

### 2.1. Wine Selection

A total of twelve white wines, commercially available, were selected for the present work (Table 1). All twelve wines, including five Swedish Solaris (Sol-SW 1/2/3/4/5) wines and seven non-Solaris, were from vintages between 2016 and 2018. All Swedish wines were produced in different municipalities from Skåne, in the South of Sweden. The non-Solaris wines included two Albariño from Spain (AL-SP (1)/(2)), three Sauvignon Blanc (two from France and one from New Zealand), one Chardonnay (from France), and one Chenin Blanc (from South Africa).

### 2.2. Sensory Analysis

Wines were evaluated blindly by 16 Swedish wine experts (11 males, 5 females), mostly local producers. Assessors were not informed about the origin of the wines they were tasting. Wines (25 mL) were evaluated in clear glasses, covered with watch glasses, labelled with three-digit codes, and presented in a randomized order. Wines were evaluated in two modalities. Firstly, typicality and quality of the wines were assessed on an unmarked linear scale (0 to 100) answering the questions: “Is this a typical Solaris wine?” (from very atypical at 0 to very typical at 100) and “rate the quality of this wine” (form very low quality at 0 to very high quality at 100). The aroma, taste, and mouthfeel of the wines were evaluated using of Check-All-That-Apply (CATA) method [37]. The data collection was carried out with the software EyeQuestion^®^ software v4 (Logic8, Woldendorp, The Netherlands).

### 2.3. Volatile Analyses

#### 2.3.1. Thiol Analyses

The level of 3-Mercaptohexanol (3MH), 3-Mercaptohexyl acetate (3MHA), 4-Methyl-4-mercaptopentan-2-one (4MMP), and furanmethanethiol (FMT) in the wines was assessed with the use of the method by Mafata, et al. (2018) [38]. The principle of the method is based on the derivatization of the thiol moiety (-SH) by 4,4′-Dithiodipyridine (DTDP), followed by sample purification and concentration by solid-phase extraction on a ENVI-C18 cartridge (Supelco Inc., Bellefonte, PA, USA), followed by analysis by UltraPerformance Convergence Chromatography coupled with tandem Mass Spectrometry (UPC2-MS/MS, Waters Viridis coupled to Xevo TQ-S triple quadrupole mass spectrometer—Waters, Milford, MA, USA) on a BEH C18 column (Waters, Milford, MA, USA). Detailed chromatographic conditions can be found in Mafata et al. (2018).

#### 2.3.2. Terpenes and Norisoprenoids

The level of terpenes and norisoprenoids was quantified in all wines following the method described by Williams and Buica [39]. In brief, 10 mL wine sample containing 3-Octanol and 2,6-Dimethyl-6-hepten-2-ol as internal standards were extracted by Headspace-Solid Phase Microextraction (HS-SPME) on a DVB/PDMS/CAR fiber from Supelco^®^ (Bellefonte, PA, USA) and injected into the Gas Chromatograph-Mass Spectrometer (GC-MS). Detailed information on the chromatographic conditions is given in the cited work [39].

#### 2.3.3. Major Volatiles

A selection of major volatiles (alcohols, esters, and acids) were analyzed using a HP-6890 Gas Chromatography-Flame Ionization Detection (GC-FID, Agilent Technologies, Waldbronn, Germany) using a high-throughput in-house method, adapted from Louw et al. [40] The sample prep consists of a liquid-liquid extraction of 5 mL sample (with 4-methyl-2-pentanol as internal standard) in 1 mL diethyl ether under sonication for 5 min. Extracts are centrifuged and the extract dried over anhydrous Na_2_SO_4_ (99%, Merck Chemicals PTY, Germiston, South Africa) before analysis by GC-FID [40].

### 2.4. Non-Volatile Analyses

#### 2.4.1. Cation Analyses

Major and minor elements of the wines were analyzed at the ICP-MS & XRF Unit of the Central Analytical Facility of the Stellenbosch University, South Africa, using a Thermo ICap 6200 ICP-AES for the major and minor elements down to mid-ppb values and an Agilent 7900 ICP-MS for the ultra-trace analysis. Data are quantified with calibration solutions prepared from NIST traceable standards, and quality control procedures according to US Environmental Protection Agency guidelines are followed to ensure accuracy of data [41].

#### 2.4.2. Organic Acids and Sugars

Sample preparation was based on the method by Eyéghé-Bickong et al. [42]. To a 200 µL of wine sample, 800 µL of deionized water was added, followed by the addition of 1 mL of internal standard stock solution (4 g/L ribitol and adipic acid) to yield a final volume of 2 mL (10× dilution). The samples were vortexed, centrifuged, and then transferred to HPLC vials for analysis. The measurement of wine organic acids, alcohols, and carbohydrates was performed using an Agilent Hi-Plx eH column (300 × 7.7 mm). Chromatographic conditions were as described in the Agilent application 5990-8264EN [43].

### 2.5. Data Analysis

The data from the sensory evaluation were analyzed with the use of XLSTAT 2019 (Addinsoft SARL, New York, NY, USA). The typicality and quality of the wines were evaluated with a two-way ANOVAs introducing the judge effect as random whereas the wine was considered a fixed effect. The consensus between judges regarding the typicality and quality of the wines was evaluated through Principal Component Analysis (PCA) and Hierarchical Cluster Analysis (HCA). The data analysis of the CATA test was performed with Correspondence Analysis (CA) on the resulting contingency table with descriptors in columns and products in rows Aroma attributes were analyzed separately from taste and mouthfeel. Chemical data were subjected to PCA and Orthogonal Partial Least Square-Discriminant Analysis (OPLS-DA) using SIMCA 16.0.1 Umetrics (Sartorium Stedim Biotech-Malmö, Sweden).

## 3. Results

### 3.1. Typicality and Quality of Swedish Solaris Wines

**Figure 1:**
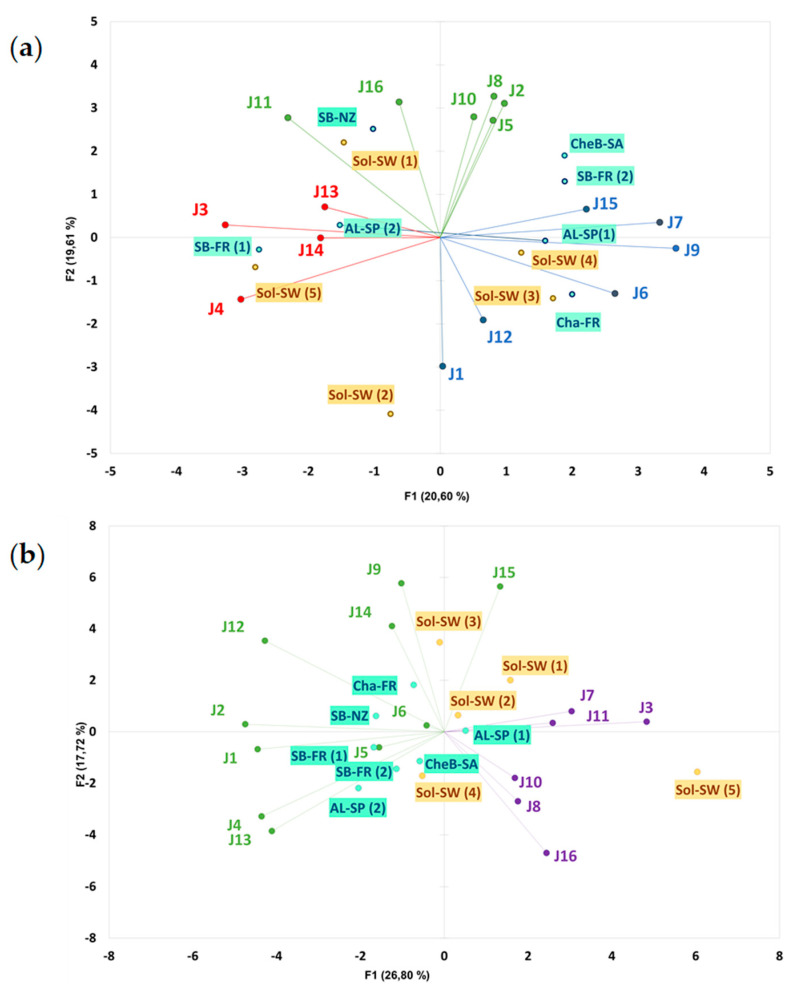
(**a**) Principal Component Analysis (PCA) Biplot representing the consensus between judges (J1-J16) according to their typicality scores. (**b**) PCA Biplot representing the consensus between judges (J1-J16) according to their quality scores.

Agreement between experts concerning typicality and quality was explored by means of PCA (Figure 1). A lack of consensus was found among the judges regarding what they perceived as a typical Solaris (Figure 1a). This lack of agreement resulted in a non-significant ANOVA result and a very close (within a range of 41–57) Solaris typicality means between samples (Supplementary Materials Table S1).

A similar result was found concerning quality ratings (Figure 1b). However, in this case the ANOVA was significant although the only significant differences were observed only between the two extreme wines.

In order to segment the panel into consensual clusters, HCA was performed on PC1 and PC2 coordinates of the typicality PCA. The HCA analysis unveiled three clusters based on the configuration of the typicality scores, typicality cluster 1 (*n* = 6, in green), typicality cluster 2 (*n* = 4, in red), and typicality cluster 3 (*n* = 6, in blue) (Figure 1a). A segmentation of the data was performed and the analysis of variance for the typicality scores was analyzed for each of the clusters. Significant differences between the wines were only found in typicality cluster 1 (Table 2). However, this cluster considered Sol-SW (1) as the most typical Solaris wine and Sol-SW (2) as the least typical. It was therefore necessary to have a look at the sensory space of these wines to have a better understanding of what was driving cluster 1 typicality scores.

As previously mentioned, significant differences were found for the quality scores of the wines, (F = 2.17; *p* = 0.018). AL-SP (1) was perceived as the wine with the highest quality (Table S1). This score was significantly higher than Sol-SW (5), wine which had the lowest quality score. The rest of the Solaris wines (Sol) were spread in the lower part of the quality rating, although no significant differences were found between these wines and the highest and lowest scoring wines. The main interest of this work was to understand the perception of Solaris wines as a group and, therefore, when looking into the wines grouped as Solaris (Sol) versus international wines (non-Sol), the latter were perceived with a significantly higher quality (t = 2.49; *p* = 0.031). As it occurred with the typicality of the wines, a disagreement between judges was also found for the perception of the wine quality (quality cluster 1, n= 10 and quality cluster 2, *n* = 6, Figure 1b). Analysis of variance of the quality was performed for each cluster. In the case of cluster 1, non-Sol wines were perceived as higher quality, with the exception of Cha-FR found at the bottom of the range (Table 2). However, Sol-SW (3) was perceived on the top range of the list. Opposite results were found for cluster 2. This group of judges perceived Sol-SW (5) as the wine with the highest quality and Sol-SW (1) was also found among the top range. Nonetheless, Sol-SW (2), (3) and (4) showed the lowest average scores in quality, together with Cha-FR.

Since typicality is a well-stablished sub dimension of wine quality [44,45], we expected a significant correlation between these two parameters. However, the regression vector (RV) coefficient between the three clusters typicality matrix and the two clusters quality matrix was low (0.349), indicating that there was no clear relationship between the typicality and quality assessments.

### 3.2. Sensory Profile of the Wines

**Figure 2:**
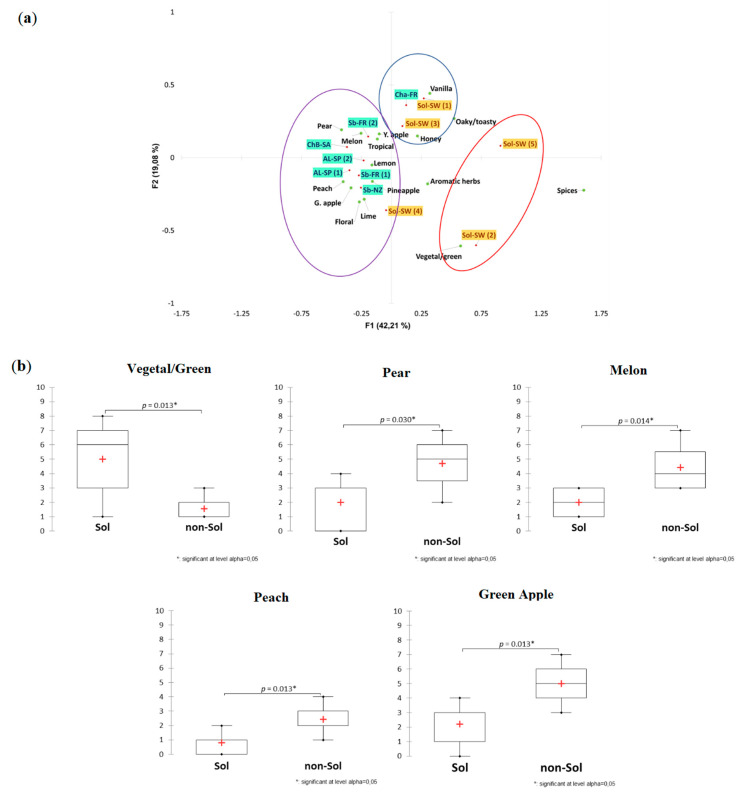
(**a**) Correspondence Analysis (CA) biplot representing the distribution of the wines according to the frequency of citation of the provided aroma descriptors. Ellipses represent the clusters found with the corresponding Hierarchical Cluster Analysis (HCA) analysis. (**b**) Box and whiskers plots illustrating the aroma attributes significantly different between Sol and non-Solaris wines based on a Student *t*-test.

**Figure 3:**
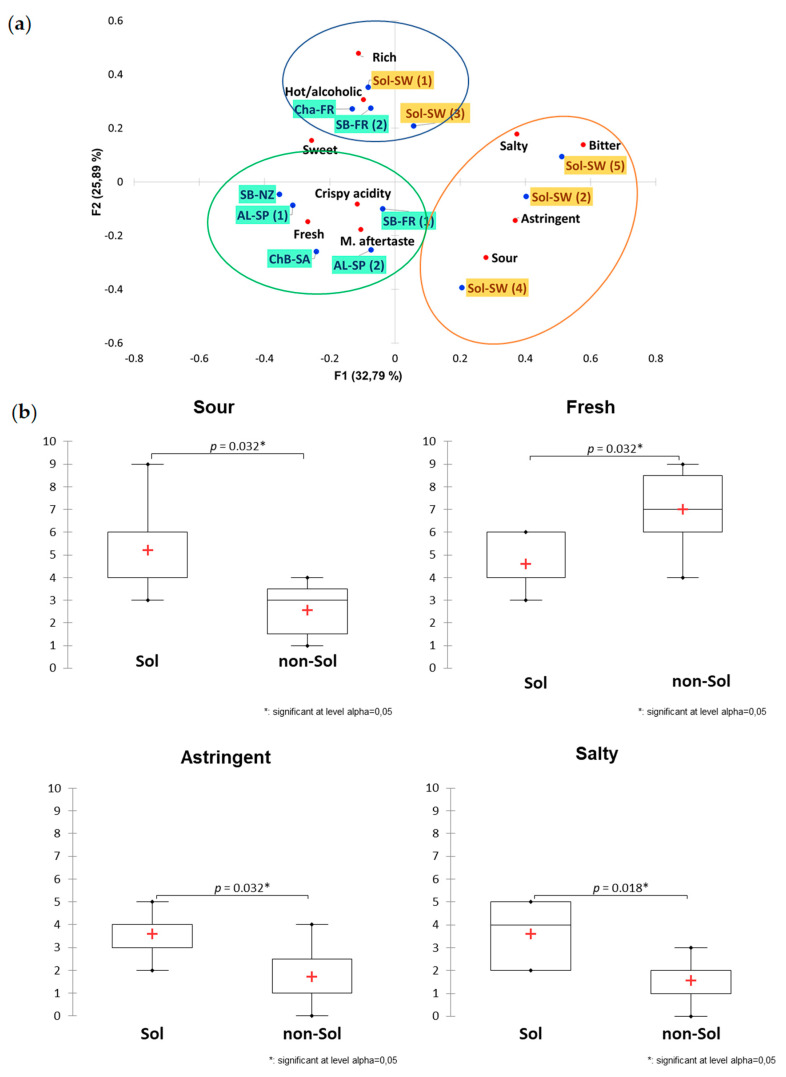
(**a**) CA biplot representing the distribution of the wines according to the frequency of citation of all the provided taste and mouth-feel descriptors. Circles represent the clusters found with the corresponding HCA analysis. (**b**) Box-and-whiskers plots illustrating the taste and mouthfeel attributes significantly different between Sol and non-Solaris wines based on a Student *t*-test.

In the aroma profile of the wines, the distribution of the different wines following the CA analysis along F1 (42.21%) was driven by the higher frequency of citation of fruity descriptors (Figure 2). Wines on the left side of F1 were more frequently described as “melon”, “peach”, “pear” and/or “pineapple”, whereas wines on the right side of F1 were characterized by “green” and aging-related descriptors. Three clusters were obtained from the corresponding HCA analysis, showing a spread distribution of Solaris wines. Sol-SW (1) and Sol-SW (3) grouped together with Cha-FR, described with ageing-related attributes such as “oaky/toasty” and “vanilla”, whereas Sol-SW (2) and Sol-SW (5) wines formed a cluster on their own, associated with “greener” aroma descriptors, such as “aromatic herbs” and “vegetal/green”. Sol-SW (4) showed a fruitier profile than the rest of Solaris wines, clustering together with most of the international wines. These results may be interpreted as three possible styles for Swedish Solaris wines: green, fruity, and oaky (Figure 3). Despite clustering together with most of the international wines, when looking at the cluster’s characteristics and linkage, Sol-SW (4) was found to be the furthest from the center of its cluster. This cluster of wines was mainly characterized by a high frequency of citation of “green apple” and “peach”.

Therefore, the Student’s *t*-test was performed to assess if any descriptors were significantly different when comparing Sol wines to non-Sol wines. The box and whisker plots in Figure 2b illustrate the descriptors which were found to be significantly different between Sol and non-Sol wines. For Sol wines, “vegetal/green” was found to be significantly higher (*p* = 0.013), whereas “peach” (*p* = 0.013), “melon” (*p* = 0.014), “pear” (*p* = 0.030), and “green apple” (*p* = 0.013) were significantly higher for non-Sol wines.

**Figure 4:**
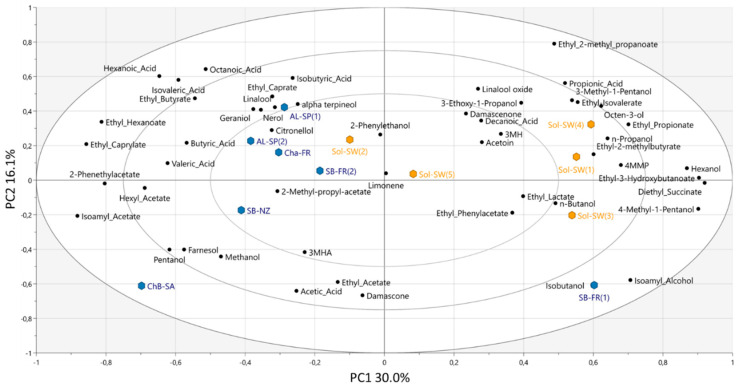
PCA biplot showing the distribution of the wines according to their volatile composition. Solaris wines are colored in orange and non-Solaris in blue.

Similar trends were observed for taste and mouthfeel (Figure 3a). Student’s *t*-tests between Sol and non-Solaris showed that non-Sol wines were perceived as fresher (Figure 3b; *p* = 0.032), whereas Sol wines were perceived as significantly saltier (*p* = 0.018), more sour (*p* = 0.032), and more astringent (*p* = 0.032) (Figure 3b). Considering the overall in-mouth description, Sol-wines were divided between two clusters. As illustrated in Figure 4, Sol-SW (1) and Sol-SW (3) grouped together in the same cluster as Cha-FR, and in this case, also with Sb-FR (1). This cluster of wines was characterized by a higher alcohol sensation and richness in mouth. Sol-SW (4) was in the same group as Sol-SW (2) and Sol-SW (5). This group of wines were more frequently cited as “bitter”, “sour”, “astringent”, and “salty”.

### 3.3. Volatile Composition

Data for volatile compounds analysis were evaluated from two different perspectives. Firstly, data were analyzed using an unsupervised approach (PCA) considering the wines as individual products. Secondly, a supervised modelling approach was performed (OPLS-DA) with two defined groups: Solaris wines (Sol) and non-Solaris wines (non-Sol). The distribution of the wines based on their aroma profile was performed including all volatile compounds data. The total explained variance of the first two component of the PCA analysis was 46.1% (Figure 4). A trend separating between most of Sol and non-Sol wines (except for Sb-FR (1)) can be observed along PC1 (30.0%). The corresponding HCA analysis showed two clusters with a spread distribution of Sol wines (Supplementary Materials Figure S1). Sol-SW (2) and Sol-SW (5) grouped together with most of international wines, whereas the second cluster, on the right side of PC1, included Sol-SW (1), Sol-SW (3), Sol-SW (4), and Sb-FR (1) (Figure 4).

**Figure 5:**
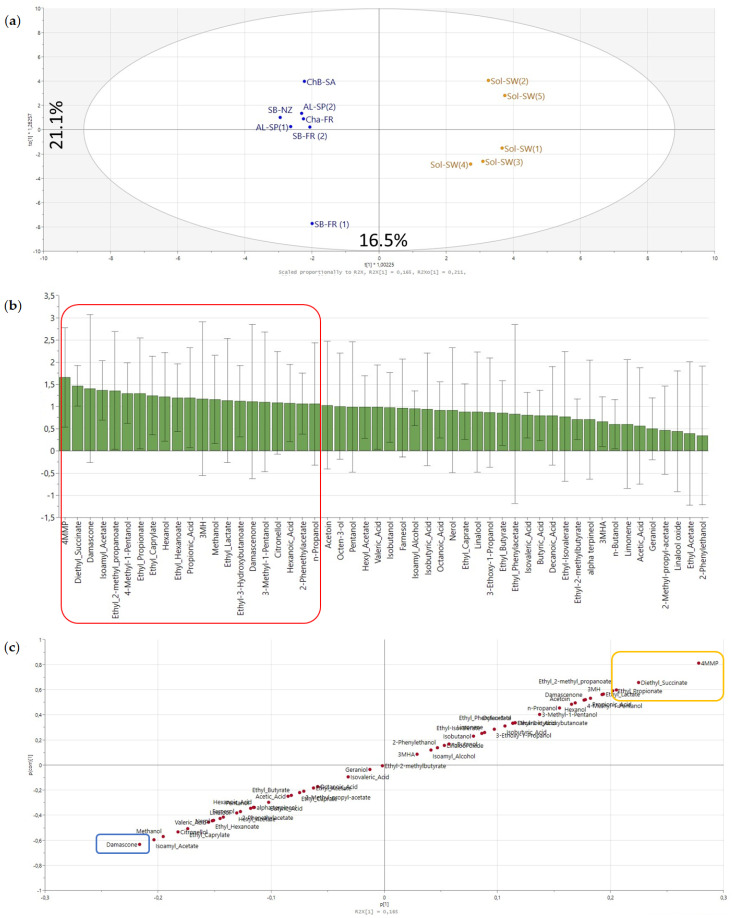
OPLS-DA score plot (**a**), VIP (Variable Importance for Projection) (**b**) and S-plot pair wise comparison (**c**) between Solaris (colored in orange) and non-Solaris wines (colored in blue) according to their volatile composition. Squares on the S-plot represent the significant variables for Sol and non-Sol based on the Student’s *t*-test.

An OPLS-DA model was built to evaluate the discriminant capacity of varietal compounds as a tool which could be a step forward in understanding the flavor potential of Solaris grapes. However, the OPLS-DA model reliability for the volatile composition was found not significant (CV-ANOVA *p*-value 0.729). This lack of significance was expected from the PCA (Figure 4) and the corresponding HCA. It has been shown in literature how model reliability is compromised when no group separation is observed on PCA scores [46]. This could be explained by a diversity in winemaking approaches within the Sol group. Indeed, OPLS-DA results showed that variability within each group was larger (F2-21.1%) than between the groups Sol and non-Sol (F1-16.5%) (Figure 5a).

As expected, it was difficult to establish a cut-off from the corresponding S-plot (Figure 5B) to determine whether some compounds were discriminating between Sol and non-Solaris wines or not. However, information extracted from the VIP (Variable Importance for the Projection) combined with a Student’s *t*-test approach [47] can help to establish a cut-off for the S-plot and determine the discriminant capacity between groups. Student’s *t*-tests were carried out on variables with a VIP value > 1. The results showed that the levels of 4-MMP, ethyl propionate (fruit-like, pineapple aroma), ethyl 2-Methyl propanoate, and diethyl succinate were significantly higher in Sol wines (located on the upper right side of the S-plot); whereas, the levels of damascone were significantly higher in non-Sol wines (lower left side of the S-plot) (Figure 5c).

The presence of varietal thiols (3-MH, 3MHA and 4MMP) was confirmed in all Sol wines. The occurrence of polyfunctional thiols has recently been reported for the first time in Solaris wines from Belgium [36]. Interestingly, 4-MMP was identified only in Sol wines in the set of wines analyzed (Supplementary Materials Table S2). This aroma compound has a characteristic smell of “box tree” and “blackcurrant”, which can also contribute to the vegetal aroma of the wines and whose levels in the wines can also be influenced by fermentative conditions [48]. Damascone, an oxygenated C13-norisoprenoid, was found to be more abundant in non-Sol wines. Damascone has been associated with a characteristic “rose”, “tea-like” and “fruity” (citrus and apple) aromas in other wine matrices [49]. Additionally, to the authors’ knowledge, this is the first time that the presence of 3-MH and 3-MHA are reported on Albariño wines (Supplementary Materials Table S2).

### 3.4. Non-Volatile Composition

**Figure 6:**
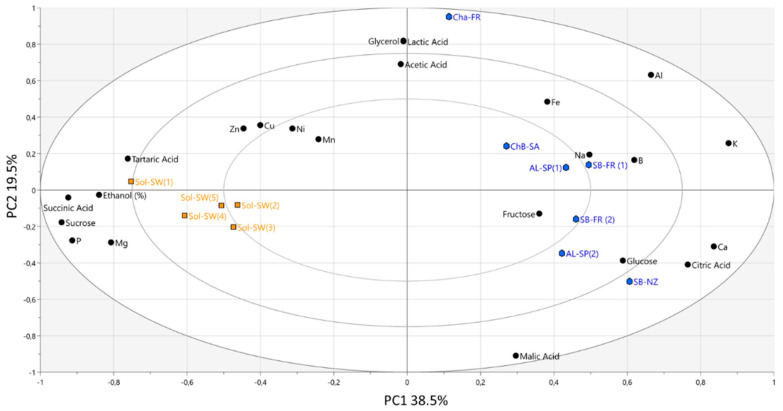
PCA biplot representing the distribution of the wines according to the non-volatile data. Solaris wines are colored in orange and non-Solaris wines are colored in blue.

The differences between Sol and non-Sol wines based on the non-volatile matrix were also investigated. The non-volatile data included basic oenological parameters such as the ethanol level of the wines (Supplementary Materials Table S3), the concentration of sugars and organic acids and the concentration of different cations and anions. As displayed in the PCA biplot in Figure 6, a clear separation between Sol and non-Sol wines was found along PC1, also representing the results from the two clusters obtained from the corresponding HCA analysis (Supplementary Materials Figure S2).

**Figure 7:**
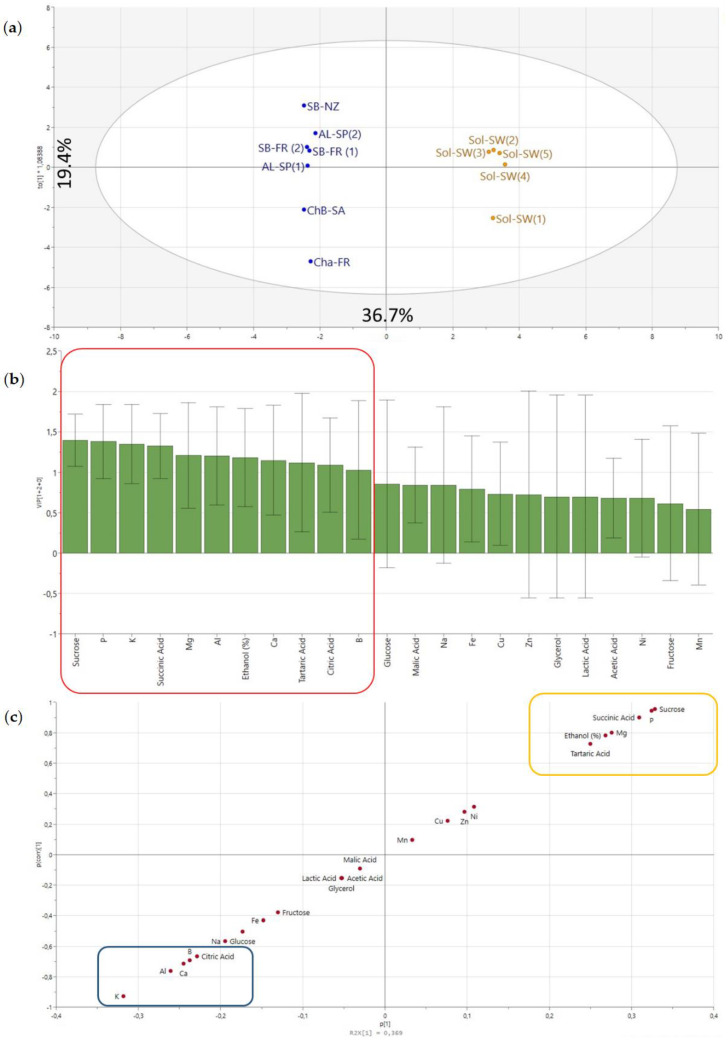
(**a**) OPLS-DA score plot. (**b**) VIP (Variable Importance for Projection). (**c**) S-plot pair wise comparison Solaris (colored in orange) vs. non-Solaris wines (colored in blue) according to the non-volatile composition. Squares on the S-plot represent the significant variables for Sol and non-Sol based on the Student *t*-Test.

The OPLS-DA model built for the non-volatile data was found to be significant (CV ANOVA *p*-value = 0.001). As illustrated in Figure 7a, and contrary to what was found for the volatile data, the differences between the Sol and non-Sol groups were larger (F1—36.7%) than within groups (F2—19.4%). Acids such as tartaric and succinic, ethanol %, and elements such as Mg and P were the components which characterized the group of Sol wines (Figure 7b). Different winemaking practices can influence the levels of some of the metabolites. The formation of succinic acid, a fermentation by-product, can be influenced by parameters such as the use of specific yeast strains, higher fermentation temperatures, or a greater level of flavonoids and lipids in the grape must [50,51].

Nonetheless, the level of succinic acid in the final wines can also be related to the grape cultivar. It has been shown that higher amounts of succinic acid are produced in *Vitis rotundifolia* during fermentation when yeast assimilable nitrogen (YAN) is provided as alanine or threonine, instead of arginine or ammonium as occurs in *Vitis vinifera* [51]. On the opposite side of the S-plot, K was shown to characterize non-Sol wines. The level of K in grapes can also influence the succinic acid formation [50].Looking at the variability within the non-Sol wines, Cha-FR was the most different to its group, most probably due to its level of lactic acid and glycerol (Figure 6).

## 4. Discussion

One of the main results of this work is the lack of a consensus regarding Solaris typicality among the Swedish wine experts. This result was contrary to previous works on varietal typicality which have shown fair consensus between French experts for Sciaccarelo wines [25] and Chardonnay and Melon de Bourgogne varietal wines [21]. A possible explanation is that French wine industry is centuries old, with well-established training oenology programs and thousands of examples of these varietal wines available for tasting. Therefore, these experts have developed a common representation of the sensory style associates to each category through extensive tasting. In contrast, the Swedish wine industry is relatively young and the number of available Solaris wines on the market is much lower. These two factors could make it more difficult for Swedish wine professionals to develop a shared sensory representation of Solaris. Moreover, the aroma profiles showed at least two different wine styles which makes prototype abstraction even more difficult for tasters. At this stage, Swedish winemakers do not have an association of a wine style to what a typical Swedish Solaris should smell and taste like.

Nevertheless, the volatile composition showed that compounds such as 4-MMP, ethyl propionate, ethyl 2-Methyl propanoate, and diethyl succinate could discriminate between Sol and non-Sol wines. A recent study has shown relevant information regarding the presence of thiol precursors in *Vitis* hybrid varieties. Despite Solaris not being included in the study, the cited work has shown that some *Vitis* hybrid grape cultivars such as SV023 and Zarya Severa, have a larger concentration of 4-MMP precursors than Sauvignon Blanc [52]. These compounds could influence the “fruity” and “green” Solaris style. The analysis of methoxypyrazines should be considered in future studies, as they are known to play a major role on the “greenness” perception the wines. A study from Van Wyngaard (2013) showed not only that 2-Methoxy-3-isobutylmethoxypyrazine (iBMP) is associated with green notes, but also that at high concentration iBMP could have a suppressive effect over the tropical aromas associated with thiols. The author also showed that at high levels, thiols could also suppress the “green notes” from iBMP [53]. Understanding the proportions between Solaris thiols and methoxypyrazines could help to understand the potential different wine styles.

Another important metabolite, at least from a health standpoint, is methanol. Previous literature pointed out that wines from fungus resistant grapes tended to show higher methanol concentrations than wines from *Vitis Vinifera* grapes [12]. In our case, no significant differences were found for methanol between Sol and non-Sol wines (t = 2.06, *p* = 0.06). Furthermore, all the samples had methanol concentrations below the OIV (Organisation Internationale de la vigne et du vin) limit for white wines (250 mg/L).

The differences in taste and mouthfeel were more obvious between Sol and non-Sol wines for both sensory evaluation and chemical composition. The bitterness and saltines associated with Sol wines could be explained by the levels of succinic acid as this organic acid has been shown to have a salty and a bitter taste [50]. Additionally, it can also play a role on the aroma as it has been shown to be involved in the formation of aroma-active esters, such as diethyl succinate [51], which was one of the discriminant aroma compounds between Sol and non-Sol. The presence of certain cations, such as Ca, K, Mg, and Na had previously been associated with wine saltiness in Mexican wines [54]. However, the salt content did not imply a higher saltiness perception in South African Chenin Blanc and Pinotage wines [55]. In the present study, Mg was the only cation found to be significantly higher in Sol wines (perceived as saltier) than non-Sol wines. However, the impact of Mg on perceived saltiness is yet to be demonstrated in a wine matrix. A study on Italian Solaris showed a large amount of (-)-epicatechin on the grapes [35]. The higher bitterness of Sol wines may be related to its presence in the wines; however, the present study did not measure the wine phenolic composition.

A higher alcohol and sucrose content was found for Sol wines. This result is consistent with previous findings that showed that Solaris grapes have a high sugar loading capacity in the berries resulting in very high brix musts [56] and very high alcohol wines [57]. Sucrose metabolism has been shown to be linked to temperature variation in plant tissues, being also a key factor for cold resistant hybrid grape cultivars such as Vidal and Beta [58]. The higher level of sucrose in Sol wines could possibly be explained by sugar addition to the musts. In cooler climate, the low temperatures may affect the sugar accumulation and, therefore, sucrose can be added to increase the alcoholic yield of the corresponding wines. As the present work does not have grape data, the significantly higher levels of sucrose in Sol wines (between two and three times higher—t = −10,821; *p* = <0.0001), may be attributed to either of the two reasons.

## 5. Conclusions

The present work has brought out relevant information regarding the sensory space and chemical characterization of Solaris wines. Sensory results have shown the possibility of different Solaris wine styles. From a chemical perspective, the present study has shown the presence of varietal compounds such as thiols, terpenes, or norisoprenoids. This study can help to have a better understanding of the flavor potential of this grape in Sweden and to develop the desired style. Future studies should include a larger set of wines and compare Solaris wines from different origins to know the full potential of the grape. Additionally, to the authors knowledge the present work has reported the presence of thiols in Albariño wines for the first time.

## Figures and Tables

**Table 1:** Wines used for the study.

Cultivar	Country	Vintage	Code
Solaris	Sweden	2016	Sol-SW (1)
Solaris	Sweden	2016	Sol-SW (2)
Solaris	Sweden	2016/2017	Sol-SW (3)
Solaris	Sweden	2016	Sol-SW (4)
Solaris	Sweden	2016	Sol-SW (5)
Albariño	Spain	2017	AL-SP (1)
Albariño	Spain	2016	AL-SP (2)
Sauvignon blanc	France	2016	SB-FR (1)
Sauvignon blanc	France	2017	SB-FR (2)
Chardonnay	France	2016	Cha-FR
Sauvignon blanc	New Zealand	2017	SB-NZ
Chenin blanc	South Africa	2018	ChB-SA

**Table 2:** Average scores for the typicality and quality clusters. Means within a column with different letters were significantly different according to Tukey test (alpha = 5%).

Typicality Cluster 1		Typicality Cluster 2		Typicality Cluster 3		Quality Cluster 1		Quality Cluster 2	
Sol-SW (1)	69.17 a	AL-SP (2)	72.82 a	Sol-SW (3)	51.23 a	SB-FR (2)	64.44 a	Sol-SW (5)	74.92 a
SB-NZ	66.52 ab	SB-FR (1)	72.30 a	AL-SP (1)	47.57 a	AL-SP (1)	62.57 a	AL-SP (1)	64.48 ab
ChB-SA	64.63 ab	Sol-SW (2)	59.87 a	ChB-SA	47.35 a	AL-SP (2)	62.12 a	ChB-SA	60.47 ab
SB-FR (2)	60.42 ab	SB-NZ	57.85 a	Sol-SW (4)	46.92 a	SB-NZ	61.63 a	Sol-SW (1)	59.03 ab
AL-SP (2)	58.82 ab	Sol-SW (4)	56.82 a	Sol-SW (2)	45.33 a	Sol-SW (3)	61.19 a	SB-FR (2)	53.98 ab
AL-SP (1)	54.88 ab	Sol-SW (5)	56.07 a	AL-SP (2)	44.80 a	ChB-SA	59.74 a	SB-FR	53.28 ab
SB-FR (1)	53.95 ab	ChB-SA	55.72 a	SB-FR (2)	43.00 a	SB-FR (1)	57.92 ab	SB-NZ	52.60 ab
Sol-SW (4)	49.70 ab	Sol-SW (1)	52.40 a	Cha-FR	41.52 a	Sol-SW (1)	55.02 ab	AL-SP (2)	50.9 5 ab
Sol-SW (5)	45.53 ab	Sol-SW (3)	52.25 a	SB-FR (1)	39.52 a	Cha-FR	53.46 ab	Sol-SW (3)	45.25 b
Sol-SW (3)	45.08 ab	Cha-FR	44.70 a	Sol-SW (1)	30.15 a	Sol-SW (4)	51.50 ab	Sol-SW (4)	45.23 b
Cha-FR	38.07 ab	SB-FR (2)	35.35 a	Sol-SW (5)	28.23 a	Sol-SW (2)	47.11 ab	Cha-FR	44.27 b
Sol-SW (2)	29.22 b	AL-SP (1)	34.17 a	SB-NZ	25.38 a	Sol-SW (5)	38.09 b	Sol-SW (2)	37.47 b

## Supplementary Materials

The following are available online at https://www.mdpi.com/2304-8158/9/8/1107/s1, Figure S1: Hierarchical Cluster Analysis for the volatile composition of the wines. Figure S2: Hierarchical Cluster Analysis for the non-volatile composition of the wines. Table S1: Average scores for the typicity and quality. Table S2: Volatile composition of the wines. Table S3: Non-volatile composition of the wines.
