# Supplementary material for: Exploring the Typicality, Sensory Space, and Chemical Composition of Swedish Solaris Wines
Source: Foods. 2020 Aug 12;9(8):1107. doi: 10.3390/foods9081107 (PMC7466253; doi:10.3390/foods9081107)
Supplement: Supplementary file 1 [file foods-09-01107-s001.pdf]

Table S1. Average scores for the typicity and quality scores for the evaluated wines. Means within a column with different letters were significantly different according to Tukey test (alpha = 5%).

Wines	Typicality	Quality
**ChB-SA**	55.93 a	60.01 ab
**AL-SP (2)**	57.06 a	57.93 ab
**AL-SP (1)**	46.96 a	63.29 a
**SB-FR (1)**	53.13 a	56.18 ab
**SB-FR (2)**	47.62 a	60.52 ab
**SB-NZ**	48.93 a	58.24 ab
**Sol-SW (1)**	50.34 a	56.53 ab
**Sol-SW (3)**	49.18 a	55.21 ab
**Sol-SW (4)**	50.44 a	49.15 ab
**Sol-SW (5)**	41.68 a	51.90 ab
**Cha-FR**	41.02 a	50.01 ab
**Sol-SW (2)**	42.93 a	43.49 b

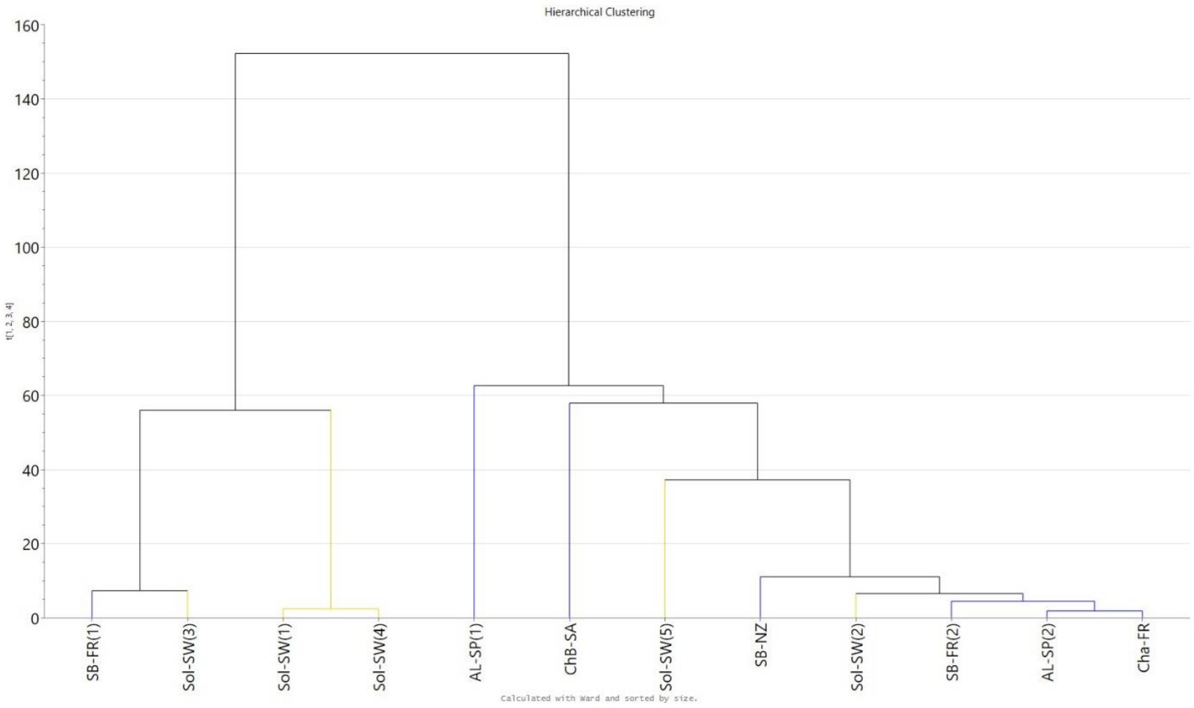

Figure S1. Hierarchical Cluster Analysis for the volatile composition of the wines. Dendrogram is coloured according to Sol wines (orange) and non-Sol wines (blue).

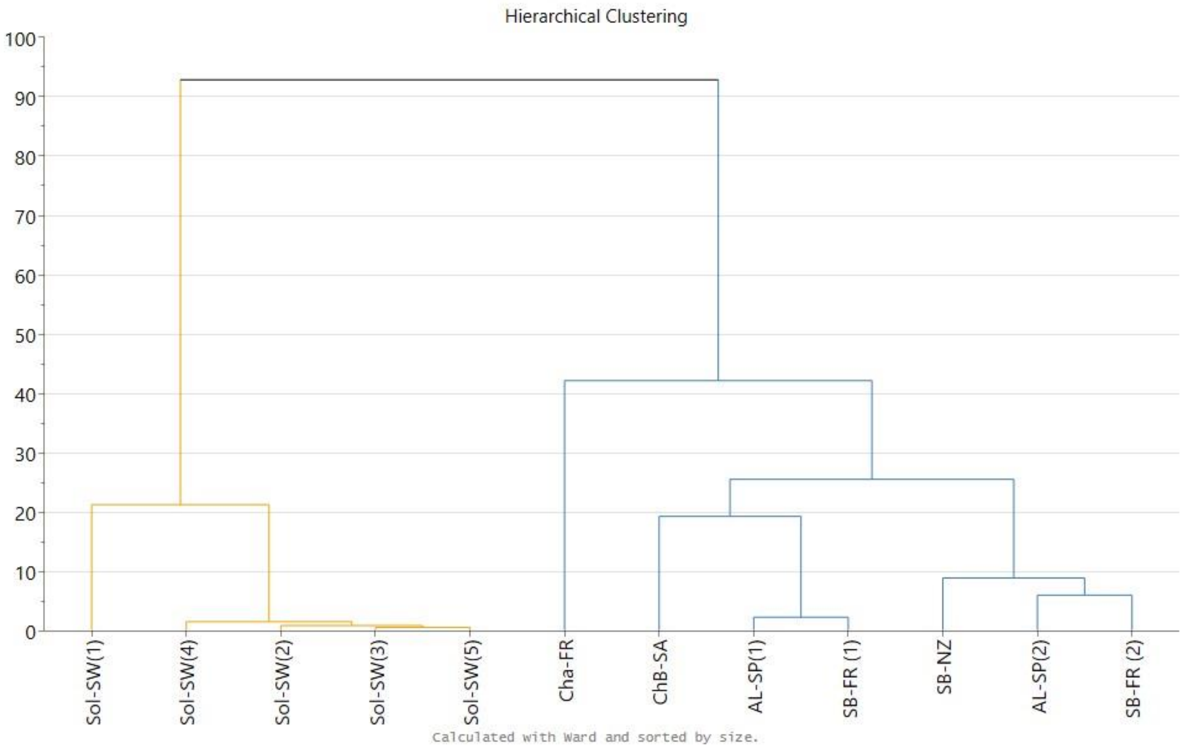

Figure S2. Hierarchical Cluster Analysis for the non-volatile composition of the wines. Dendrogram is coloured according to Sol wines (orange) and non-Sol wines (blue).

Table S2. Volatile composition of the wines.

Coding data	Sol-SW(1)	Sol-SW(2)	Sol-SW(3)	Sol-SW(4)	Sol-SW(5)	AL-SP (1)	AL-SP(2)	Cha-FR	ChB-SA	SB-FR (2)	SB-FR (1)	SB-NZ
3MH	528	154	133	143	284	55	20	181	47	198	50	195
3MHA	4.65	2.8	nd	nd	nd	nd	0.65	nd	7.9	nd	nd	nd
4MMP	8	2.7	12	5	4	nq	nq	nq	nq	nq	nq	nq
Limonene	17.96	18.46	17.23	18.33	20.80	17.33	17.56	18.85	17.38	17.33	18.18	19.57
Linalool oxide	13.99	10.88	17.48	21.68	12.26	32.92	5.24	10.82	1.82	5.80	8.40	16.01
Linalool	4.79	1.38	1.79	0.98	0.52	61.55	21.58	4.02	4.33	6.00	3.52	6.07
alpha terpineol	7.24	4.85	5.10	3.99	6.05	80.86	14.63	6.29	<LOD	3.98	3.48	18.05
Citronellol	5.88	4.62	5.44	5.08	4.34	10.59	6.25	5.62	5.99	6.81	5.45	5.91
Nerol	nd	nd	nd	nd	nd	3.62	0.98	nd	0.24	0.47	nd	0.52
Damascone	1.66	1.64	1.58	1.91	1.36	1.96	1.70	1.87	2.36	1.93	2.78	2.08
Damascenone	2.55	0.99	1.28	0.99	2.68	1.58	1.60	1.09	0.89	1.50	0.68	0.09
Geraniol	9.37	17.04	8.86	8.44	19.79	22.64	13.76	11.10	11.39	11.83	10.32	10.95
Farnesol	<LOD	<LOD	<LOD	nd	<LOD	<LOD	1.41	<LOD	3.56	2.18	<LOD	<LOD
Ethyl_Acetate	55.95	39.06	38.69	49.35	58.11	35.18	41.52	50.49	63.88	55.92	56.15	63.63
Methanol	34.84	35.96	36.40	45.24	44.46	39.42	37.00	55.42	55.65	45.57	46.81	62.87
Ethyl_Propionate	0.42	0.21	0.15	0.51	0.15	0.12	0.12	0.14	0.10	0.17	0.20	0.09
Ethyl_2-methyl_propanoate	0.11	0.11	0.06	0.15	0.09	0.09	0.08	0.08	<LOQ	0.05	0.05	0.06
2-Methyl-propyl-acetate	0.00	0.04	0.01	0.00	0.02	0.00	0.03	0.01	<LOQ	0.03	0.02	0.06
Ethyl_Butyrate	0.23	0.28	0.24	0.31	0.23	0.25	0.31	0.35	0.28	0.34	0.14	0.29
n-Propanol	104.99	24.20	40.85	148.11	22.25	20.30	32.23	27.38	28.55	43.14	41.96	25.86
Ethyl-2-methylbutyrate	0.03	0.02	0.02	0.03	0.03	0.02	0.03	0.02	<LOD	0.02	0.04	0.03
Ethyl-Isovalerate	0.05	0.05	0.04	0.07	<LOD	0.03	0.04	0.04	<LOD	0.03	0.04	0.03
Isobutanol	20.23	23.85	36.07	20.00	21.93	14.01	17.46	2.43	19.98	17.75	47.04	20.19
Isoamyl_Acetate	0.38	1.38	0.23	0.41	0.69	0.81	2.01	1.46	2.83	1.63	0.18	2.95
n-Butanol	0.76	0.64	0.85	1.22	0.75	0.50	0.73	0.76	0.78	1.08	0.93	0.67
Isoamyl_Alcohol	155.24	133.61	171.87	148.52	126.94	122.02	139.62	128.06	137.93	143.10	195.63	136.22
Ethyl_Hexanoate	0.46	0.54	0.36	0.62	0.60	0.64	0.81	0.83	0.79	0.74	0.20	0.70
Pentanol	0.07	0.10	0.06	0.07	0.05	0.10	0.08	0.07	0.24	0.10	0.06	0.08
Hexyl_Acetate	0.06	0.13	0.02	0.09	0.05	0.07	0.08	0.07	0.17	0.16	0.02	0.15
Acetoin	1.47	0.98	1.08	21.92	27.43	1.23	2.46	2.51	2.00	2.81	1.69	1.97

4-Methyl-1-Pentanol	0.01	0.01	0.02	0.02	0.01	0.00	0.00	0.00	<LOQ	0.01	0.02	0.00
3-Methyl-1-Pentanol	0.05	0.04	0.06	0.07	0.06	0.03	0.04	0.07	<LOQ	0.06	0.04	0.04
Ethyl_Lactate	26.14	20.74	51.29	31.76	139.26	10.61	10.40	12.57	9.38	18.30	46.90	9.51
Hexanol	2.45	1.72	3.11	3.62	1.41	2.16	0.81	0.86	0.92	2.02	2.79	0.95
3-Ethoxy-1-Propanol	8.90	0.87	0.62	9.36	1.00	1.61	3.17	3.29	1.13	5.27	0.72	1.32
Ethyl_Caprylate	0.14	0.26	0.21	0.18	0.57	0.63	0.59	0.49	0.57	0.51	0.06	0.47
Octen-3-ol	0.02	0.01	0.01	0.02	0.01	0.01	0.00	0.02	<LOD	0.01	0.01	0.00
Acetic_Acid	313.62	229.92	224.78	265.41	403.04	226.13	232.88	312.74	425.52	342.19	361.70	408.66
Ethyl-3-Hydroxybutanoate	0.57	0.28	0.80	0.80	0.33	0.48	0.34	0.31	<LOD	0.31	0.88	0.29
Propionic_Acid	2.05	1.30	1.03	2.23	0.91	1.07	0.83	0.59	0.36	1.78	0.57	0.81
Isobutyric_Acid	0.56	1.01	0.54	0.76	1.09	0.84	0.86	0.66	0.57	0.59	0.44	0.71
Butyric_Acid	1.18	1.34	1.43	1.50	1.18	1.42	1.58	1.82	1.78	1.67	0.89	1.19
Ethyl_Caprate	0.03	0.05	0.03	0.04	0.08	0.04	0.09	0.08	<LOD	0.09	0.02	0.08
Isovaleric_Acid	0.60	0.91	0.36	0.61	0.57	0.77	0.66	0.64	0.65	0.67	0.36	0.65
Diethyl_Succinate	9.75	2.15	5.98	10.44	7.36	2.13	1.24	1.80	0.90	1.38	8.73	0.81
Valeric_Acid	0.02	0.04	0.03	0.05	0.01	0.04	0.08	0.08	0.07	0.01	0.01	0.07
Ethyl_Phenylacetate	0.00	0.00	0.06	0.00	0.00	0.00	0.00	0.00	<LOQ	0.00	0.00	0.00
2-Phenethylacetate	0.01	0.34	0.00	0.01	0.05	0.14	0.16	0.14	0.27	0.12	0.00	0.31
Hexanoic_Acid	3.87	4.57	3.12	5.16	4.22	5.09	6.72	6.24	4.72	5.67	1.82	4.75
2-Phenylethanol	11.50	41.44	16.99	13.93	16.51	30.25	14.02	15.33	12.07	16.10	22.84	12.48
Octanoic_Acid	4.85	7.11	3.51	6.04	5.49	5.21	9.08	7.75	4.55	7.78	2.16	5.65
Decanoic_Acid	1.90	1.09	0.23	1.17	0.64	0.44	1.25	0.11	0.38	1.13	0.62	0.84

Thiols are reported in ng/L. Terpenes, norisoprenoids and major volatiles are reported in μg/L. nd - not detected, nq – not quantifiable. The limit of detection (LOD) and limit of quantitation (LOQ).

Table S3. Non-volatile composition of the wines.

Coding data	Sol-SW(1)	Sol-SW(2)	Sol-SW(3)	Sol-SW(4)	Sol-SW(5)	AL-SP(1)	AL-SP(2)	Cha-FR	ChB-SA	SB-FR (1)	SB-FR (2)	SB-NZ
Citric Acid	0.27	0.25	0.28	0.25	0.25	0.48	0.53	0.17	0.38	0.47	0.54	0.4
Tartaric Acid	5.68	5.96	5.27	4.91	6.62	4.46	2.22	3.79	5.6	4	2.48	2.83
Malic Acid	3.29	3.45	4.25	3.99	3.4	4.66	5.61	0.58	2.79	3.73	4.11	5.9
Succinic Acid	2.51	1.66	2.01	2.1	2.01	1.19	1	1.43	0.95	1.3	1.14	1.2
Lactic Acid	0.31	0.56	0.3	0.25	0.45	0.21	0.23	2.36	0.22	0.35	0.36	0.21
Acetic Acid	0.26	0.19	0.15	0.29	0.29	0.33	0	0.43	0.48	0.2	0.24	0.22
Sucrose	1.35	1	1.14	1.02	1.38	0.43	0.43	0.47	0.47	0.45	0.47	0.46
Glucose	0.3	0.24	0.15	0.2	0.15	0.19	0.38	0.29	0.24	0.38	0.49	0.92
Fructose	1.81	0.71	0.88	1.18	0.73	1.52	0.75	1.24	1.14	1.06	2.1	2.22
Glycerol	0.4	0.66	0.39	0.34	0.54	0.3	0.32	2.51	0.31	0.44	0.45	0.29
Ethanol	16.2	14.2	14.3	14.6	13.9	13	12.2	12.3	13.2	12.5	13.8	11.2
Al	0.16	0.19	0.05	0.07	0.09	1.03	0.38	0.93	0.6	0.94	0.61	0.16
B	2.42	1.55	2.97	1.95	1.30	3.38	2.28	2.68	4.3	2.93	3.88	2.90
Ca	44.27	62.23	60.28	53.37	67.39	79.50	88.66	59.78	69.9	74.49	72.04	79.18
Cu	0.07	0.03	0.07	0.10	0.04	0.03	0.07	0.09	0.1	0.01	0.02	0.02
Fe	0.83	0.50	0.20	0.21	0.72	1.71	0.58	1.01	0.9	2.16	0.42	0.24
K	549.70	491.60	643.80	510.20	572.50	764.10	746.70	765.70	744.1	718.30	696.60	737.50
Mg	105.70	98.48	103.00	95.47	97.21	80.27	80.31	69.07	92.1	55.62	68.12	80.94
Mn	1.79	0.88	0.53	0.44	0.77	1.16	1.22	0.78	1.1	0.74	0.50	0.09
Na	17.10	10.04	8.35	7.79	12.12	34.05	12.54	16.61	56.0	22.46	10.85	31.69
Ni	0.01	0.02	0.02	0.02	0.01	0.02	0.01	0.02	0.0	0.02	0.01	0.00
P	318.30	279.10	379.00	412.20	290.10	139.90	131.60	114.30	142.0	81.33	127.60	139.10
Zn	2.42	0.53	0.62	0.65	0.50	0.68	0.46	0.99	0.6	0.80	0.76	0.12

Sugars and organic acids are expressed in g/L. Ethanol content is expressed in %. Cations and anions from elemental analysis are expressed in mg/L.
